# Micro-CT and Histomorphometric Study of Bone Regeneration Effect with Autogenous Tooth Biomaterial Enriched with Platelet-Rich Fibrin in an Animal Model

**DOI:** 10.1155/2021/6656791

**Published:** 2021-05-11

**Authors:** Yoon-Ki Lee, Puneet Wadhwa, HongXin Cai, Sung-Uk Jung, Bing Cheng Zhao, Jae-Suk Rim, Dong-Hyuck Kim, Hyon-Seok Jang, Eui-Seok Lee

**Affiliations:** ^1^Department of Oral and Maxillofacial Surgery, Graduate School of Clinical Dentistry, Korea University, Seoul 08308, Republic of Korea; ^2^The CONVERSATIONALIST Club, School of Stomatology, Shandong First Medical University & Shandong Academy of Medical Sciences, Tai'an, Shandong 271016, China

## Abstract

The aim of this study was to evaluate the potential of tooth biomaterials as bone graft biomaterials for bone healing in rabbits. We prepared tooth biomaterial and platelet-rich fibrin (PRF) to fill the round-shaped defect in the skull of New Zealand white rabbits. These cranial defects were treated with different conditions as follows: group 1, a mixture of tooth biomaterials and platelet-rich fibrin (PRF); group 2, only tooth biomaterials; group 3, only PRF; and group 4, the unfilled control group. Specimens of the filled sites were harvested for analysis with microscopic computerized tomography (micro-CT) and histomorphology at 4 and 8 weeks. As a result of micro-CT, at 4 weeks, the bone volume percentages in groups 1 and 2 were 50.33 ± 6.35 and 57.74 ± 3.13, respectively, and that in the unfilled control group was 42.20 ± 10.53 (*p* = 0.001). At 8 weeks, the bone volume percentages in groups 1 and 2 were 53.73 ± 9.60 and 54.56 ± 8.44, respectively, and that in the unfilled control group was 37.86 ± 7.66 (*p* = 0.002). The difference between the experimental group 3 and the unfilled control group was not statistically significant. Histomorphologically, the total new bone was statistically different.

## 1. Introduction

In order to fill the damaged tissue caused by trauma or disease, people use various bone graft materials, such as bovine bone, allogeneic bone, and hydroxyapatite. Due to its reliable bone regeneration ability, autogenous bone is considered an ideal bone graft material. Although autogenous bone grafts have many advantages, they also have some shortcomings. They require additional surgery and have some limitations, such as morbidity at the donor site, perioperative pain, and infection [1]. Therefore, allogenic bone and xenogenic bone have been used as a substitute instead of autogenous bone grafts in clinical practice [2, 3]. Trends in resorption, widespread disease transmission, and relative monetary cost are the main disadvantages of allografts and xenografts [4, 5]. Due to the advantages of synthetic bone, hydroxyapatite (HA) and tricalcium phosphate (TCP) have been widely studied and used as bone substitutes [6, 7]. Although synthetic bone exhibits lower immunotoxicological reactions as compared to allogenic bone, its bone repairing ability is lacking in comparison to the autogenous bone grafts [8].

Materials derived from patients' own autogenous blood can replace bone as graft materials for bone defects, reducing the incidence of donor sites and operating time. Platelet-rich plasma (PRP) has been a classic example of them. The use of PRP alone and in fusion with other biomaterials is feasible. However, PRP preparation needs chemical additives and goes through several steps [9, 10]. Platelet-rich fibrin (PRF) is a second-generation platelet derivative that offers some advantages over PRP; it does not require additives and can be prepared in one step by slow centrifugation [11]. PRF consists of the fibrin matrix as opposed to the jellifying agents and bovine clotting factors in PRP which helps in reducing the incidence of coagulopathies. In the case of PRP growth factors, growth factors are released for a shorter time period whereas PRF releases growth factors in a slow and sustained manner [12]. Fibrin is a derivative of fibrinogen, a plasma molecule. The fibrillary molecule present in plasma helps to coagulate platelets during hemostasis [13]. Thrombin acts on fibrinogen to form the fibrin monomer, which has the ability to polymerize with other fibrin monomer molecules to form fibrin fibers [14]. In the rabbit tibia with bone defects, the addition of ceramic biomaterials and fibrin sealants promoted bone regeneration [15]. Fibrin adhesive is always a good choice to maintain large-scale bone graft fragments [16]. However, fibrin without bone graft material not only fails to assist bone formation but also has an inhibitory effect on healing [17]. This fibrin inhibits the binding of bone to some biomaterials [18]. Therefore, care must be taken when selecting biological materials that bind to fibrin.

Tooth biomaterials are composed of hydroxyapatite (HA), amorphous calcium phosphate (ACP), octacalcium phosphate (OCP), and small amounts of *β*-tricalcium phosphate (*β*-TCP), which are derived from teeth. However, the crystallinity and amount of hydroxyapatite are highly related to the part of the tooth. Hydroxyapatite is able to be degraded with enzymes and absorbed *in vivo* [19]. Among the organic components, the enamel consists mainly of enamel proteins [20]. The dentin consists of collagen and noncollagenous proteins. Type I collagen is found dominantly in the collagen matrix [21].

The tooth biomaterial has good biocompatibility and is used as a scaffold material to help bone defect repair. Tooth biomaterials serve as scaffolds to deliver induced cells to the healing site and provide cues for controlling the structure of newly formed tissues [22]. This dental biomaterial degrades slowly and has excellent mechanical properties and biocompatibility [19]. Since the tooth biomaterial has organic and inorganic components, it is an ideal scaffold material and can be used as a PRF scaffold material. This study explores the potential of tooth biomaterials mixed with PRF *in vivo* as bone graft materials in bone formation.

## 2. Materials and Methods

The study protocol was approved by the Korea University Institutional Animal Care and Use Committee (KUIACUC-2010-195). The management of all animals and the surgery protocol were approved by the committee.

### 2.1. Animal Groups

New Zealand white rabbits (3 months old) weighing averagely 2.3 kg (range 2.0–2.5 kg) were kept in separate cages. They were fed a standard diet and allowed to freely mobilize during the study. All animals were treated in compliance with the Care and Use of Animals policy of the committee guidelines.

### 2.2. Tooth Biomaterial

The tooth-derived biomaterial was produced by the Korea Tooth Bone Bank (Seoul, Korea) for this experiment. The tooth biomaterial was made from extracted teeth, and it was in powder form.

### 2.3. Platelet-Rich Fibrin (PRF)

A blood sample (10 mL) was procured through the ear vein of the rabbit, and centrifugation was done for 12 minutes at a rate of 400 *g*. After the blood was centrifuged, it was separated into three different layers, and the middle layer representing PRF was taken.

### 2.4. Study Design

In this study, 20 New Zealand white rabbits were selected with bilateral defects made on both sides of the calvarial bone (diameter 8.0 mm). The tooth biomaterial was made from extracted teeth. The groups (*n* = 10) were divided as follows. The critical size defect was filled randomly with a combination of a graft of the tooth biomaterial and PRF (group 1), tooth biomaterial only (group 2), or PRF only (group 3). An empty defect served as the control group (group 4). After 4 and 8 weeks, animals were sacrificed, and specimens were prepared for histological and histomorphometric analysis.

### 2.5. Animal Surgery

**Figure 1:**
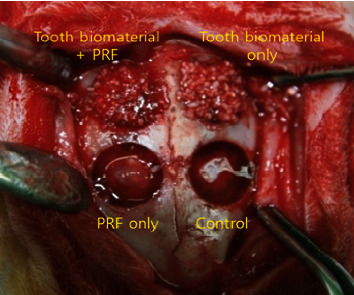
Experiment design. A combination of tooth biomaterial and platelet-rich fibrin was placed on the experimental side, and the control defect was left unfilled. The tooth powder only and PRF only were grafted into the other bony defects.

The experimental protocols for this animal model were previously described [19, 20]. In the calvarial bones of each animal, bilateral full-thickness calvarial defects were created. The rabbits were anesthetized by intramuscular injection of a combination of 0.3 mL xylazine (10 mg/kg, Rompun; Bayer Korea Ltd., Seoul, Korea) and 0.4 mL ketamine hydrochloride (100 mg/mL, Ketara; Yuhan, Seoul, Korea). The cranium area was then shaved and disinfected with povidone-iodine. A 3 cm longitudinal incision was made over the calvarium from the nasal bone to the occipital protuberance. After a midline incision was created in the periosteum, soft tissue was dissected, and the bone was exposed by gentle retraction of the periosteum. The calvarial bones were exposed when sharp subperiosteal dissection reflected the pericranium from the outer table of the cranial vault. Four 8 mm diameter defects were created with a trephine bur under irrigation with 0.9% sterile saline solution. We chose 8 mm defects in our study as they are suitable for comparison of different materials or groups; all groups under investigation can be used in a single animal, thereby avoiding individual variations [23]. The tooth biomaterial mixed with PRF, tooth biomaterial, and PRF were placed on the bony defect, respectively, and the control group was not filled (Figure 1). Then, the skin and pericranium were closed over the defect with 3-0 silk. Following the surgery, each rabbit was individually caged and received food and water. Postoperatively, the rabbits received antibiotic gentamicin (1 mg/kg) (Kookje, Seoul, Korea) intramuscularly 3 times daily for 3 days for prophylaxis to prevent wound infection after surgery. 4 mg/kg carprofen was administered for pain relief.

### 2.6. Sacrifice and Qualitative Evaluation

Ten animals were sacrificed at 4 and 8 weeks postoperatively. A 4-week period is suitable for studying the early period of healing response, whereas at 8 weeks, late healing like bone incorporation and bone graft resorption can be assessed [23]. The specimens were removed from the cranium with a round bur carefully, and any signs of inflammation were examined grossly. After microscopic computerized tomographic (*μ*-CT) examination, specimens were fixed in 10% buffered formalin solution before proceeding with the histological preparations.

### 2.7. Microscopic Computerized Tomography

**Figure 2:**
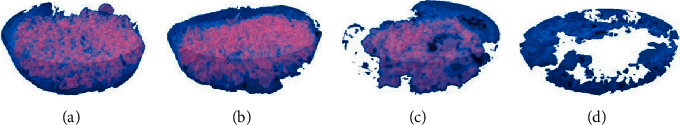
Microscopic computerized tomography at 4 weeks. The defect size was smaller in the experimental groups 1, 2, and 3 than in the control group at 4 weeks. (a) Defect size in group 1. (b) Defect size in group 2. (c) Defect size in group 3. (d) Defect size in group 4. Most bony defects were filled by new bone at 8 weeks.

The prepared specimens were assessed by micro-CT using Skyscan 1172 micro-CT (Skyscan, Belgium). Following the calibration, the specimen was scanned in sections of 0.05 mm thickness. The scanned image was reconstructed with CT-volume software (Skyscan, Belgium; Figure 2). The calibrated 3-dimensional image was shown in the gross profiles of the specimens. Image analysis of the structural morphometric parameters of the micro-CT was performed with CT-analyzer software (Skyscan, Belgium). Since the shape of the initial defect is round with a diameter of 8.0 mm, the region of interest (ROI) is regarded as the size and shape of the initial defect surrounding the bone graft area. The threshold of the bone standard is 25%, which is recommended by the manufacturer. The region of interest for the specimen was evaluated for percentage bone volume (PBV, %), bone volume (BV, mm^3^), and bone surface density (bone surface/bone volume, mm^2^/mm^3^). The bone volume content and density were calculated using the software.

Bone volume (BV, mm^3^) is the volume of the part segmented as bone.

Percentage bone volume (PBV, %) is the ratio of the segmented bone volume to the total volume in the region.

Bone surface density (bone surface/bone volume, mm^2^/mm^3^) is the ratio of the segmented bone surface to the total volume in the region.

### 2.8. Histomorphometric Evaluation

**Figure 3:**
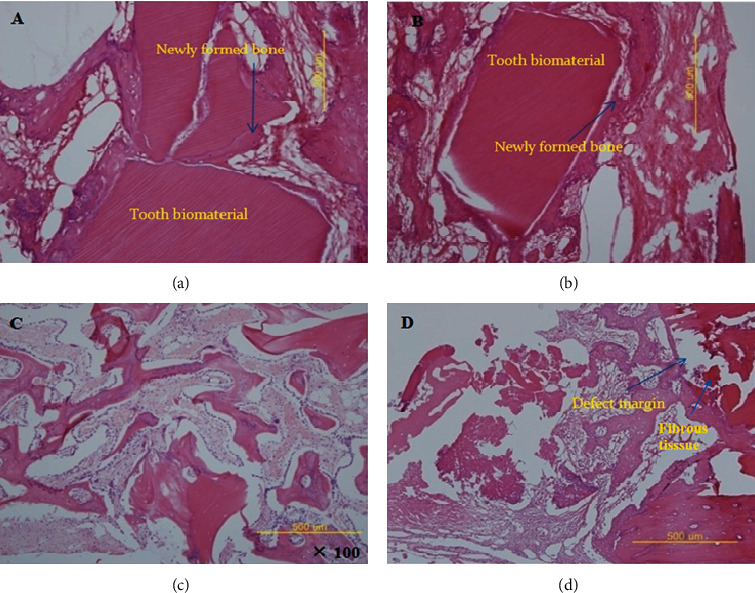
Histologic findings (H&E staining) at 4 weeks. (a) Augmented areas are constructed by newly formed bone bridges and covered with new bone. New bone formation around the tooth biomaterial (group 1). (b) The bone defect is almost completely filled by the remnants of the tooth biomaterial (group 2). (c) Magnified image of the experimental group 3 (PRF only) showed new bone formation. (d) A control group specimen healed primarily with a fibrous band of tissue.

After radiographic micro-CT analysis, each specimen underwent dehydration and embedding. Specimens were fixed in 10% neutral buffered formalin, dehydrated in ethanol, and decalcified using 5% formic acid (Fisher Diagnostics, Fair Lawn, NJ, USA) for 2 weeks. Two fragments were both embedded to show the sagittal section in the paraffin block. Tissue blocks were cut into at 4 *μ*m, and staining was done using hematoxylin and eosin stain. Selective digital images of sections were taken using a digital camera (DP-20; Olympus, Tokyo, Japan; Figure 3). Sections from the center of each block were examined by light microscopy, and images were captured (ImageScope Version 9.1.19.1571; Aperio Technologies, CA, USA). The images were analyzed by SigmaScan Pro (SPSS, Chicago, IL). The amount of new bone as a percentage of the bone defect area was calculated.

### 2.9. Statistical Analysis

The data was tested for equal variance and normality. Micro-CT data was compared using the Kruskal-Wallis test and Mann-Whitney test and histomorphometric data by ANOVA by ranks. After a significant result, groups were compared using the Mann-Whitney test. A paired-sample *t*-test was used to determine statistical significance between groups. *p* values of <0.05 were considered to be statistically significant. A paired *t*-test was used for comparison of the samples from the same animal. The statistically significant level was set as *p* < 0.05.

## 3. Results

### 3.1. Microscopic Computerized Tomography

The results of the *μ*-CT analysis are depicted in Table 1. The average value of all measured variables was higher in experimental groups 1 and 2 than in the control group at 4 weeks following surgery. There was a statistically significant difference in the PBV value. The PBV in experimental groups 1 and 2 at 4 weeks after the operation was 50.33 ± 6.35 and 57.74 ± 3.13, respectively, and that in the control group was 42.20 ± 10.53 (*p* = 0.001). The PBV in experimental groups 1 and 2 at 8 weeks after the operation was 53.73 ± 9.60 and 54.56 ± 8.44, respectively, and that in the control group was 37.86 ± 7.66 (*p* = 0.002). The size of the remaining defect was larger in the control group in comparison with the experimental group (Figure 2). The other variables did not show a statistically significant difference (*p* > 0.05).

### 3.2. Histomorphometry

**Figure 4:**
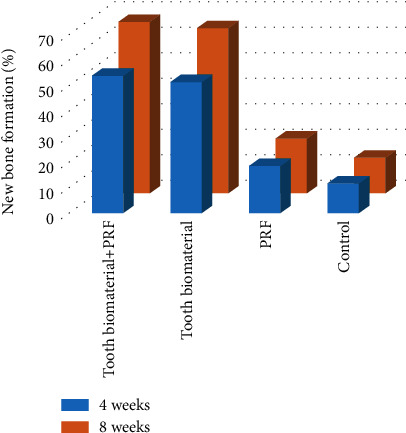
Result of histomorphometric analysis (total new bone formation) at 4 and 8 weeks.

The results of histomorphometry showed that the total new bone was 11.57 ± 15.12% in the control group at 4 weeks following surgery. It was 18.45 ± 17.34% in group 3, 51.56 ± 26.45% in group 2, and 53.87 ± 27.60% in group 1 at 4 weeks after the operation (Figure 4). The total new bone was 14.21 ± 23.83% in the control group at 8 weeks after the operation. It was 18.45 ± 17.25% in experimental group 3, 64.96 ± 20.59% in experimental group 2, and 67.45 ± 21.50% in experimental group 1 at 8 weeks after the operation (Figure 4). The difference was statistically significant (*p* = 0.021).

## 4. Discussion

There are many studies regarding bone healing in rabbit calvarial defects. Humber et al. reported that bone ceramic material with a polyethylene glycol hydrogel membrane was the most effective, and the healing of bone defects without membrane support was affected [24]. Pripatnanont et al. studied the effect of PRF on bone formation when added to deproteinized bovine bone and autogenous bone [25]. The addition of PRF enhanced bone formation more when using autogenous bone, but there was no significant effect on osteogenesis when mixed with deproteinized bovine bone. PRF is a good choice as a covering membrane and graft support material in the bony defect, but PRF cannot always improve bone formation significantly.

In this study, in order to promote the restoration of bony defects, the tooth biomaterial was used as a bone graft material with PRF. PRF when used without bone graft material not only fails to assist bone formation but also has an inhibitory effect on healing [17], so PRF combined with the tooth biomaterial can be considered a combinational bone graft material. The tooth biomaterial produced from extracted teeth has been studied as an effective bone graft material [19]. The advantages of the tooth biomaterial are low price, slow degradation rate, plasticity, and good biocompatibility [19]. These advantages are significantly beneficial to connective tissue regeneration. However, some features like slow degradation may adversely affect bone regeneration. In order for bone regeneration to be successful, inflammation and degradation velocity need to be controlled. To overcome the limitations of teeth as bone substitute materials, their structure has also been modified when preparing tooth biomaterial.

PRF is an autologous product which minimizes the risk of cross infections and immunologic reactions. The alpha granule of the platelet has many growth factors. The alpha granule of the platelet releases growth factors which are transforming growth factor (TGF), platelet-derived growth factor (PDGF), vascular endothelial growth factor (VEGF), epidermal growth factor (EGF), and insulin-like growth factor-1 (IGF-1). These growth factors play a fundamental role in the initial healing mechanism. According to the 3-dimensional shape of defects, PRF has to be used as a barrier membrane or a pouch of the grafting materials. Using tooth biomaterials as a combined template with PRF, CT results showed that the new bone volume of experimental groups 1 and 2 was significantly higher than that of the control group. The percentage bone volume was also significantly higher in experimental groups 1 and 2 than in the control group. These results were further confirmed by histomorphometric analysis (Figure 4).

Histomorphometric analysis further confirmed the results. The results of this study also showed that the two analyses are closely related. The tooth biomaterial is a macromolecule that can be degraded *in vivo* (Figure 3(b)); although the degradation time of the tooth biomaterial is reported differently, it may take a few years to lose its shape completely. During resorption, the tooth biomaterial does not induce an immunogenic or foreign body reaction mediated by lymphocytes and multinucleated giant cells. The tooth biomaterial was used in this study, and it was difficult to detect the presence of immunogenicity in the histologic examination. The tooth biomaterial seemed not to be resorbed within several months. The tooth biomaterial has osteoconductive ability and acts as a scaffold for bone regeneration. The application of the presented product might be limited to cavity-formed deformities because it was granular. Rapid resorption of the graft material may not induce new bone regeneration, whereas slow resorption may enhance bone regeneration and osteoconduction. The biodegradability of the bone graft material might be controlled by the size and quality of the fragment [26]. However, the relationship between osteoconductive bone formation effect and biodegradability is still unclear. In the future, the biodegradation of the tooth biomaterial will continue to be studied. In summary, a combination of PRF with the tooth biomaterial showed a faster bone healing effect compared to the unfilled control group. The tooth biomaterial could be used for the reconstruction of bony defects as a new substitute. However, results from this study cannot provide the significant bone formation ability of PRF, although PRF has important cytokines involved in initial healing. This is similar to the results of an earlier study where PRF, when used alone in a peri-implant bone defect for bone regeneration, did not show any significant difference from the control group [27]. Another study with noncritical size defects in the rabbit tibia did not observe any significant enhanced effects in healing dynamics with the use of leucocyte PRF [28]. Manipulation of PRF might influence its application. Future studies with an accurate application of tooth biomaterial with PRF as a tissue engineering technique will be needed.

## 5. Conclusions

In summary, a combination of PRF with the tooth biomaterial showed a faster bone healing effect compared to the unfilled control group. The tooth biomaterial could be used for the reconstruction of bony defects as a new substitute. However, results from this study cannot provide the significant bone formation ability of PRF, although PRF has important cytokines involved in initial healing. Manipulation of PRF might influence its application. Future studies with an accurate application of tooth biomaterial with PRF as a tissue engineering technique will be needed.

## Figures and Tables

**Table 1:** Micro-CT analysis.

4 weeks	Tooth biomaterial+PRF	Tooth biomaterial	PRF	Control	*p* value

Bone volume (mm^3^)	15.68 ± 8.37	18.14 ± 9.49	11.11 ± 6.99	11.91 ± 8.24	NS
Percentage bone volume (%)	50.33 ± 6.35	57.74 ± 3.13	35.70 ± 13.54	42.20 ± 10.53	0.001
Bone surface density (mm^2^/mm^3^)	24.65 ± 2.77	20.64 ± 3.10	34.49 ± 11.08	27.29 ± 8.60	0.004

8 weeks	Tooth biomaterial+PRF	Tooth biomaterial	PRF	Control	*p* value

Bone volume (mm^3^)	15.73 ± 9.46	17.23 ± 9.66	13.89 ± 7.14	15.05 ± 7.98	NS
Percentage bone volume (%)	53.73 ± 9.60	54.56 ± 8.44	39.29 ± 6.17	37.86 ± 7.66	0.002
Bone surface density (mm^2^/mm^3^)	21.74 ± 5.05	21.90 ± 4.75	26.45 ± 4.39	30.45 ± 10.78	NS

PRF = platelet-rich fibrin; NS = not significant.

## Data Availability

The data used to support the findings of this study are available from the corresponding author upon request.
